# Pruning-Based Sparse Recovery for Electrocardiogram Reconstruction from Compressed Measurements

**DOI:** 10.3390/s17010105

**Published:** 2017-01-07

**Authors:** Jaeseok Lee, Kyungsoo Kim, Ji-Woong Choi

**Affiliations:** Department of Information & Communication Engineering, Daegu Gyeongbuk Institute of Science and Technology (DGIST), Daegu 771-813, Korea; jayslee@dgist.ac.kr (J.L.); ssi09@dgist.ac.kr (K.K.)

**Keywords:** biomedical signal processing, electrocardiogram, compressed sensing, sparse signal recovery, tree pruning

## Abstract

Due to the necessity of the low-power implementation of newly-developed electrocardiogram (ECG) sensors, exact ECG data reconstruction from the compressed measurements has received much attention in recent years. Our interest lies in improving the compression ratio (CR), as well as the ECG reconstruction performance of the sparse signal recovery. To this end, we propose a sparse signal reconstruction method by pruning-based tree search, which attempts to choose the globally-optimal solution by minimizing the cost function. In order to achieve low complexity for the real-time implementation, we employ a novel pruning strategy to avoid exhaustive tree search. Through the restricted isometry property (RIP)-based analysis, we show that the exact recovery condition of our approach is more relaxed than any of the existing methods. Through the simulations, we demonstrate that the proposed approach outperforms the existing sparse recovery methods for ECG reconstruction.

## 1. Introduction

It is well known that electrocardiogram (ECG) sensors enable effective medical diagnosis for heart problems, such as arrhythmia and myocardial infarction, in everyday life [1,2,3]. In this regard, implanted ECG-based pacemakers and wearable ECG monitoring devices were developed to detect critical problems in the cardiovascular system [4]. Meanwhile, recently-developed electrocardiogram (ECG) sensors in everyday life require stable and long time capability for developing wearable devices in ambulatory environments [5,6]. Due to the growing demand of smart wearable devices, the major issue for recent ECG sensors is to achieve efficient management of large quantities of real-time biosignals in ambulatory environments [7]. As a means of ECG signal processing implemented with low power and small data storage, one of the promising paradigms that has received much attention recently is the compressed sensing (CS)-based signal compression and reconstruction [8,9,10,11,12,13,14,15,16,17,18,19,20,21,22,23,24]. The well-known finding of the CS-based data reconstruction is that signals can be recovered from far fewer measurements than traditional schemes whenever the signal is sparse (signals with a very small number of nonzero coefficients, that is, s is a *K*-sparse signal if ${∥s∥}_{0}=K\ll \dim\left(s\right)$, which can be exactly reconstructed from underdetermined measurement y=Φs) and the sensing mechanism satisfies the restricted isometry property (RIP), which is given as follows.

**Definition** **1.** *The sensing matrix $\Phi \in R^{M\times N}$ (M≪N) is said to satisfy the RIP of order K if there exists a restricted isometry constant (RIC) $\delta_{K}\in \left(0,1\right)$, such that:*

$$\sqrt{1-\delta_{K}}{∥s∥}_{2}\leq {∥\Phi s∥}_{2}\leq \sqrt{1+\delta_{K}}{∥s∥}_{2}$$

*for any K-sparse vector $s\in R^{N}$.*

**Figure 1:**
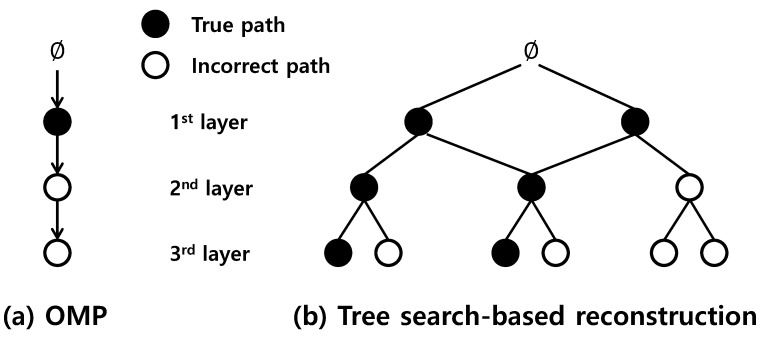
Illustration of conventional orthogonal matching pursuit (OMP) and recovery based on tree search where the true paths contain only elements in the support *T*, and incorrect paths consist of at least one element from $T^{c}$. While OMP does not provide any further chances after it selects an incorrect index in the second layer (**a**), multiple path investigation in (**b**) enables a reduction in the misdetection of the support element.

Additional benefits of the CS-based ECG processing are (1) the computationally-efficient data compression and (2) the guarantee of exact reconstruction from far fewer measurements than conventional methods. While the computational efficiency of data compression can be easily demonstrated since it requires only simple linear matrix multiplication (see Definition 1), our main interest lies in the reduction in the number of required measurements ensuring exact sparse signal recovery from compressed ECG data. A popular method for identifying the sparest signal s from the measurements y=Φs is to formulate the $\ell_{0}$-minimization problem as:

(1) $$\begin{array}{c} \min_{s}{∥s∥}_{0}\;\mathrm{subject}\;\mathrm{to}\;y=\Phi s \end{array}$$

where $\Phi \in R^{M\times N}$ (M≪N) is often called the sensing matrix. Since Equation (1) is known to be NP-hard [9], $\ell_{1}$-relaxation methods, such as basis pursuit (BP) [9], BP denoising (BPDN) [10] (or Lasso [11]) and the Dantzig selector [12], were introduced. Other than $\ell_{1}$-relaxation methods, further reduction in complexity can be achieved by the greedy approaches. To be specific, greedy algorithms attempt to identify the support $T=\{j\;|\;1\leq j\leq N,\;s_{j}\neq 0\}$ (index set of nonzero entries of s) in an iterative fashion, returning a sequence of estimates of the sparse input vector. However, although the greedy algorithm, such as orthogonal matching pursuit (OMP) [21], enables computationally-efficient implementation, its performance in general is not quite satisfactory, in particular in the presence of noise. This is mainly due to the fact that stepwise identification of the support elements might lead to a myopic decision in each iteration. Moreover, such a criterion does not provide any further chances to correct the mistake of selecting incorrect (i.e., *j*, such that $s_{j}=0$) indices once selected [21,22,23] (see Figure 1a).

The aim of this work is to introduce a new sparse signal recovery scheme that overcomes such drawbacks of conventional methods and achieves effective ECG reconstruction. By employing the tree search with an aggressive pruning strategy, our method achieves not only accurate ECG reconstruction, but also real-time implementation suitable for ambulatory environments. The main benefit of the tree search is that it enables multiple candidate investigations for identifying the support $T=\{j\;|\;1\leq j\leq N,\;s_{j}\neq 0\}$ (see Figure 1b). That is, since the tree search examines the reliability of multiple index sets simultaneously, it improves the reconstruction performance by reducing the misdetection, as well as the false alarm probabilities (the misdetection and the false alarm probabilities in this manuscript denote the probabilities of the support index not being selected and the incorrect index ($j\in T^{c}$) being identified as the support, respectively). In fact, many of the previous works in the literature focused on recovering sparse signals using the tree structure [16,17,18,19,20]. For instance, tree search-based orthogonal matching pursuit (TB-OMP) constructs the search tree by spreading multiple branches for each path [16], and its modified version was introduced in [17]. In fast Bayesian matching pursuit (FBMP), a fixed number of paths with the best posterior probabilities survives in each layer [18]. Further, the multipath matching pursuit (MMP) [19] attempts to select multiple branches (L≥2) by choosing maximally-correlated indices with the residual, and the combined method of A${}^{*}$ search [25,26] and orthogonal matching pursuit (OMP) [21] was introduced as a stage-wise residual minimization employing an effective pruning strategy [20].

Our approach, referred to as tree pruning-based matching pursuit (TPMP), provides further improvement by exploiting the full dictionary information with aggressive pruning strategies for each path. To be specific, the proposed TPMP considerably reduces the computational burden of the brute-force tree search, yet achieves excellent recovery performance by jointly implementing two pruning criteria, that is (1) the pre-scanning and (2) the pruning-based tree search. In the pre-scanning stage, we greedily choose a small number of promising column indices of the sensing matrix. If we denote the set of column indices obtained in the pre-scanning stage as Θ, then we set K≤|Θ|≪N where |Θ| is the cardinality of Θ. Once the pre-scanning is finished, the search tree is initialized by spreading the paths using only elements of Θ, so that the number of total possible paths in the tree is reduced from NK to |Θ|K, where ab=a!b!(a−b)!. For additional alleviation of the computational burden, TPMP employs a pruning strategy for removing unpromising paths from the tree. Similar to sphere decoding (SD) or list sphere decoding (LSD) with probabilistic pruning criteria [27,28,29,30], the pruning strategy is based on computing the cost function by greedily estimating the further path. Instead of obtaining the probabilistic characteristics of each path as previous works, our method exploits a full-blown candidate with cardinality *K*, which is constructed by combining the current path and greedily estimating further indices considering the complete dictionary information. By doing so, TPMP reduces the possibility of not selecting the support element (as well as selecting the incorrect index) subject to the constraint of sparsity level *K*. In addition, we demonstrate that this can rather reduce the running time complexity by shutting down the search in the early stage of the search tree.

While the preliminary version of this work was presented for an arbitrary system in [31], we show that the proposed method is highly suitable for ECG processing with some modifications and performs close to the best possible estimator (the estimator referred to as the oracle least squares (LS) estimator where the support information is given) [32]. To be specific, we reduced the cardinality of Θ for constructing smaller number of paths in the search tree for real-time implementation. In addition, we modified the cost function computation to maintain high reconstruction accuracy since investigating a smaller number of paths might degrade the performance. In order to achieve further reduction in complexity, we also employ a new stopping criterion with marginal performance loss by limiting the minimum pruning threshold. Moreover, compared to [31], we demonstrate that such modifications not only reduce the search complexity, but also improve the exact recovery condition (ERC) bound. From numerical simulations, we show that our proposed method outperforms the existing methods with practical complexity and provides additional flexibility for hardware implementation.

The rest of this manuscript is organized as follows. In Section 2, we briefly provide our setup for compressing and reconstructing ECG and then propose the TPMP algorithm. In Section 3, we analyze the exact recovery condition under which TPMP identifies the support accurately. In Section 4, we provide the numerical performance of the proposed method and then conclude in Section 5.

## 2. Tree Search-Based ECG Reconstruction

**Figure 2:**
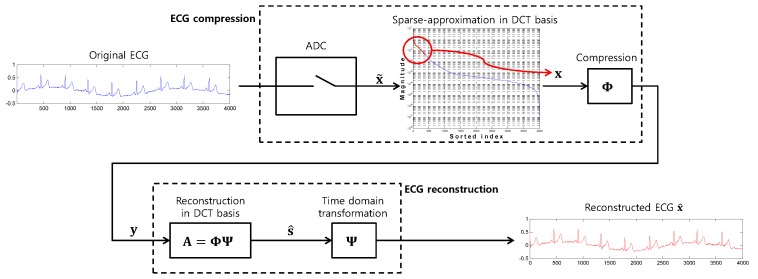
Basic structure of ECG compression and reconstruction. Note that the reconstruction is based on the discrete cosine transform (DCT) basis ($\hat{s}$), while compression is performed in the time domain (y=Φx).

In this section, we introduce a low-power ECG reconstruction method based on the tree search where the system model is provided in Figure 2. We first introduce an existing ECG compression procedure following the compressed sensing-based system architecture (Section 2.1) and then discuss our proposed method for reconstructing the ECG data from compressed measurements (Section 2.2).

### 2.1. ECG Compression

The digitized signal $\tilde{x}$ of the original ECG is approximated into x by selecting only *K* dominant elements of $\tilde{s}=\Psi^{-1}\tilde{x}$ where **Ψ** is the N×N discrete cosine transform (DCT) basis matrix. In other words, $\tilde{s}$ can be approximated as a *K*-sparse signal s with negligible information loss as:

(2) $$\begin{array}{c} \tilde{x}=\Psi \tilde{s}\approx x=\Psi s. \end{array}$$

After that, x is compressed into $y\in R^{M\times N}$ (M≪N) as:

(3) $$\begin{array}{c} y=\Phi x+v=\Phi \Psi s+v=As+v=\sum_{j\in T}a_{j}s_{j}+v \end{array}$$

where $\Phi \in R^{M\times N}$ is the sensing matrix (or compression matrix), A=ΦΨ, $a_{j}$ and $s_{j}$ are the *j*-th column of A and the *j*-th entry of s, respectively, and $v\in R^{M}$ is the additive noise (while the sparse structure in the DCT domain is still preserved, the noise v denotes the measurement distortion of y after the compression procedure or during the transmission process). Note that since |T|=K≪N, CS-based compression offers the linear superposition of *K* elements of s and, thus, enables its implementation with a substantially small number of digital architectures. From the measurement reduction perspective, it is worthwhile noting that the support information at the compression stage cannot be jointly provided to the reconstruction part. That is, for *T* to be given at the reconstruction stage, the information amount to be delivered increases from dim(y)=M to dim(y)+|T|=M+K (dim(y) is the dimension of y), which is against our intention. Furthermore, since the compression is based on the approximated signal s with a sparse structure in the DCT basis, the sensing matrix should then obey the restricted isometry property (RIP) given as:

$$\sqrt{1-\delta_{K}}{∥s∥}_{2}\leq {∥\Phi \Psi s∥}_{2}={∥\mathrm{As}∥}_{2}\leq \sqrt{1+\delta_{K}}{∥s∥}_{2}.$$

Considering such property, one of the good choices for the sensing matrix **Φ** is a random matrix, since such a matrix is said to obey the RIP with high probability [33].

### 2.2. ECG Reconstruction via Tree Search

**Figure 3:**
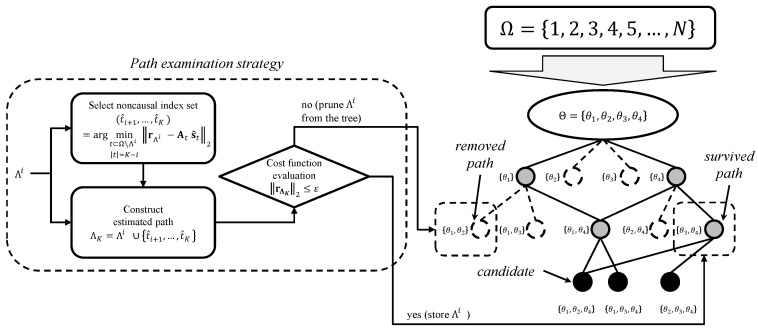
Illustration of the proposed method when |Θ|=4 and K=3. The branches of each node consist of only the elements in Θ, and the paths with large cost functions are removed from the search.

In order to achieve low complexity ECG reconstruction with improved recovery accuracy, two key pruning criteria of our method are the pre-scanning and the pruning-based tree search. In the pre-scanning stage, we greedily choose the columns of A=ΦΨ that are highly likely to be associated with nonzero entries of the sparse vector. In other words, the pre-scanning reduces the index set to be investigated from Ω={1,2,⋯,N} to a small subset Θ of Ω (i.e., Θ⊂Ω and |Θ|≪N). Then, the tree search is performed by using only the elements of Θ (see Figure 3). While any existing sparse recovery algorithm can be used to obtain Θ, we use a simple method for complexity reduction by choosing only *K* indices in this work. That is, we select *K* column indices of A=ΦΨ corresponding to the columns with maximum correlation in magnitude with y as:

(4) $$\begin{array}{c} \Theta =\arg\max_{|I_{K}|=K}{∥A_{I_{K}}^{\prime }y∥}_{2} \end{array}$$

where $I_{K}$ is an arbitrary subset of Ω with cardinality *K* and $A_{I_{K}}$ is the submatrix of A containing the columns associated with the indices in $I_{K}$. Note that since Θ is constructed by simply choosing *K* indices corresponding to maximally correlated columns with y, the computational burden in the first stage is nearly negligible.

Once the pre-scanning is finished, a pruning-based tree search is performed to select the index set that minimizes the cost function. In this stage, an aggressive pruning strategy is employed to remove the paths with a small possibility of being the support (index set of nonzero entries). As shown in Figure 3, only the paths that are not removed in the *i*-th layer spread branches in the (i+1)-th layer. The pruning strategy is based on removing paths with a larger cost function than the pruning threshold *ϵ*, since such paths have little hope to be the support. In the beginning of the search, the initial pruning threshold *ϵ* can be determined as any positive number, since the cost function of the support *T* is zero ($∥r_{T}{∥}_{2}=0$) and thus the true path can survive as long as *T* is found at least once. In order to compute the cost function $J\left(\Lambda^{i}\right)={∥y-A_{\Lambda^{i}}{\hat{s}}_{\Lambda^{i}}∥}_{2}$ of the path $\Lambda^{i}=\left\{t_{1},\;t_{2},\;\cdots ,\;t_{i}\right\}$, we obtain the temporarily required indices following the current path, the so-called posterior indices. By doing so, proposed TPMP greedily obtains the remaining part of each path $\Lambda^{i}$ and estimates its residual in magnitude at the end of the search (i.e., bottom layer). To this end, the posterior index set $\left\{{\hat{t}}_{i+1},\;{\hat{t}}_{i+2},\;\cdots ,\;{\hat{t}}_{K}\right\}$ of each path is temporarily chosen where ${\hat{t}}_{\ell }$ (i+1≤ℓ≤K) are highly likely to be the support among the elements of $\Theta \setminus \Lambda^{i}$. In fact, a similar concept of estimating the residual magnitude when the search is completed was proposed in [20]. While [20] presented three cost models to directly estimate the residual in magnitude (for example, when using the multiplicative cost model, the estimated residual in magnitude at the bottom layer is determined by the multiplication of a constant *α* and residual magnitude of the current path, i.e., $\alpha ∥r_{\Lambda^{i}}{∥}_{2}$), we focus on obtaining actual child node of each path. This problem is yet another problem of reconstructing the (K−i)-sparse signal, and in fact, the proper choice of the algorithm enables sufficient reconstruction accuracy with practical computational complexity (in our numerical simulations, we used the subspace pursuit (SP) algorithm). For instance, one can attempt to find $\left\{{\hat{t}}_{i+1},\;\cdots ,\;{\hat{t}}_{K}\right\}$ minimizing the residual in magnitude:

(5) $$\begin{array}{c} \left\{{\hat{t}}_{i+1},\;\cdots ,\;{\hat{t}}_{K}\right\}=\arg\underset{|I|=K-i}{\min_{I\subset \Omega \setminus \Lambda^{i},}}{∥r_{\Lambda^{i}}-A_{I}{\hat{s}}_{I}∥}_{2} \end{array}$$

where $r_{\Lambda^{i}}=y-A_{\Lambda^{i}}{\hat{s}}_{\Lambda^{i}}$, ${\hat{s}}_{\Lambda^{i}}=A_{\Lambda^{i}}^{†}r_{\Lambda^{i}}$ and $A_{\Lambda^{i}}^{†}={\left(A_{\Lambda^{i}}^{\prime }A_{\Lambda^{i}}\right)}^{-1}A_{\Lambda^{i}}^{\prime }$. To be specific, the posterior indices $\left\{{\hat{t}}_{i+1},\;\cdots ,\;{\hat{t}}_{K}\right\}$ in Equation (5) can be obtained by MMP [19], where this choice is to pursue accurate estimation of the cost function. On the other hand, other greedy methods, such as orthogonal matching pursuit (OMP) [21] or subspace pursuit (SP) [23], can be also used for simpler hardware implementations.

After the posterior index set is obtained, the cost function of $\Lambda^{i}$ is then computed using $\Lambda_{K}=\Lambda^{i}\cup \left\{{\hat{t}}_{i+1},\;\cdots ,\;{\hat{t}}_{K}\right\}$ (note that the cost function is computed by using the candidate with cardinality *K*). That is, if the $\ell_{2}$-norm of the residual is greater than the threshold *ϵ* ($∥r_{\Lambda_{K}}{∥}_{2}>\epsilon$), then the path is removed and whenever the search of a layer is finished and $∥r_{\Lambda_{K}}{∥}_{2}$ is replaced as the newly updated *ϵ*. The construction of the posterior index set by using the existing greedy method might be computationally burdensome if a nontrivial number of paths exist in the tree. Therefore, we attempt to additionally alleviate such search complexity by employing the stopping criteria, which constrains the minimum residual in magnitude by ${cE[∥v∥}_{2}^{2}]=cN\sigma^{2}$ for some non-negative constant 0≤c≤1. In fact, although we assumed *c* to satisfy 0≤c≤1 since $∥r_{T}{∥}_{2}=∥P_{T}^{⊥}{v∥}_{2}\leq {∥v∥}_{2}$, if a larger error tolerance is acceptable, *c* can be assumed to be any proper positive constant larger than 1. In the noise-free scenario (v=0), the initial pruning threshold *ϵ* can be determined as any positive number. This is because since $∥r_{T}{∥}_{2}=0$ for noiseless y, any positive *ϵ* is larger than the cost function of the support *T* (set of nonzero elements of the sparse vector) and thus the true path can survive as long as $\Lambda_{K}$ is obtained as *T* at least once. Therefore, if we set c=0 and whenever any path satisfying $r_{\Lambda_{K}}=0$ is found, then we regard $\Lambda_{K}$ as the support and immediately shut down the search. On the other hand, in the noisy scenario (v≠0), we assume a positive *c* (0<c), since $∥r_{T}{∥}_{2}={∥P_{T}^{⊥}v∥}_{2}>0$. Note that from the accurate reconstruction perspective, too aggressive pruning should be avoided, and thus, small *c* should be assumed, and vice versa for complexity reduction. Through performance guarantee analyses in Section 3 and numerical simulations in Section 4, we demonstrate that this stopping criteria not only improves the recovery performance as well as the condition bound, but also achieves substantial reduction in search complexity. We summarize the proposed TPMP algorithm in Table 1.

## 3. Recovery Bound for Exact Reconstruction

In this section, we provide the sufficient condition under which TPMP accurately reconstructs the *K*-sparse signal s. In our analysis, we assume that the posterior index set of each path is constructed by MMP to show how maximally our bound can be relaxed.

The following lemmas are useful for our analysis.

**Lemma** **1.** (Lemma 3 in [8]): If the sensing matrix A satisfies the RIP of both orders $K_{1}$ and $K_{2}$, then $\delta_{K_{1}}<\delta_{K_{2}}$ for any $K_{1}<K_{2}$.

**Lemma** **2.** *(Consequences of RIP [8,22]): If $0<\delta_{\left|I\right|}<1$ exists for I⊂Ω, then for any vector $x\in R^{\left|I\right|}$,*

$$\begin{array}{c} \left(1-\delta_{\left|I\right|}\right){∥s∥}_{2}\leq ∥A_{I}^{\prime }A_{I}{s∥}_{2}\leq \left(1+\delta_{\left|I\right|}\right){∥s∥}_{2},\\ \frac{1}{1+\delta_{\left|I\right|}}{∥s∥}_{2}\leq ∥{\left(A_{I}^{\prime }A_{I}\right)}^{-1}{s∥}_{2}\leq \frac{1}{1-\delta_{\left|I\right|}}{∥s∥}_{2}. \end{array}$$

**Lemma** **3.** *(Lemma 2.1 in [34]): Let $I_{1},\;I_{2}\subset \Omega$ and $I_{1}\cap I_{2}=\emptyset$. If $0<\delta_{|I_{1}\left|+\right|I_{2}|}<1$ exists, then:*

$$\begin{array}{c} ∥A_{I_{1}}^{\prime }A_{I_{2}}{s∥}_{2}\leq \delta_{|I_{1}\left|+\right|I_{2}|}{∥s∥}_{2}. \end{array}$$

**Lemma** **4.** *For M×N matrix A, ${∥A∥}_{2}$ is bounded as:*

(6) $$\begin{array}{c} {∥A∥}_{2}\leq \sqrt{1+\delta_{\min(M,N)}} \end{array}$$

**Definition** **2.** *(Residual definition in [35]): For the index set $\Lambda^{i}$ with cardinality $|\Lambda^{i}|=i$, the residual $r_{\Lambda^{i}}$ is rewritten as:*

(7) $$\begin{array}{ccc} r_{\Lambda^{i}} & = & P_{\Lambda^{i}}^{⊥}y=y-A_{\Lambda^{i}}{\hat{s}}_{\Lambda^{i}}\\ & = & y-A_{\Lambda^{i}}{\left(A_{\Lambda^{i}}^{\prime }A_{\Lambda^{i}}\right)}^{-1}A_{\Lambda^{i}}^{\prime }y\\ & = & A_{T\setminus \Lambda^{i}}s_{T\setminus \Lambda^{i}}-A_{\Lambda^{i}}{\left(A_{\Lambda^{i}}^{\prime }A_{\Lambda^{i}}\right)}^{-1}A_{\Lambda^{i}}^{\prime }A_{T\setminus \Lambda^{i}}s_{T\setminus \Lambda^{i}}\\ & = & A_{T\setminus \Lambda^{i}}s_{T\setminus \Lambda^{i}}-A_{\Lambda^{i}}z_{\Lambda^{i}}\\ & = & A_{T\cup \Lambda^{i}}{\bar{s}}_{T\cup \Lambda^{i}} \end{array}$$

*where $P_{\Lambda^{i}}^{⊥}=I-A_{\Lambda^{i}}{\left(A_{\Lambda^{i}}^{\prime }A_{\Lambda^{i}}\right)}^{-1}A_{\Lambda^{i}}^{\prime }$ is the projection matrix onto the orthogonal complement of $span(\Lambda^{i})$, $z_{\Lambda^{i}}={\left(A_{\Lambda^{i}}^{\prime }A_{\Lambda^{i}}\right)}^{-1}A_{\Lambda^{i}}^{\prime }A_{T\setminus \Lambda^{i}}s_{T\setminus \Lambda^{i}}$ and ${\bar{s}}_{T\cup \Lambda^{i}}={\left[s_{T\setminus \Lambda^{i}}-z_{\Lambda^{i}}\right]}^{\prime }$.*

### 3.1. Exact Recovery from Noiseless Measurements

TPMP is guaranteed to exactly reconstruct s if the following two conditions are jointly satisfied:

(3-1) (Theorem 1) At least one support index should be found in the pre-scanning (i.e., T∩Θ≠∅).
(3-2) (Theorem 2) At least one true path $\Lambda^{i}\subset T$ has to survive the pruning strategy in each layer.

If (3-1) holds, then at least one branch in each layer of the tree is the support element. Therefore, whenever there is at least one true path in current layer that is not removed by the pruning strategy, (3-1) enables the true path to proceed further. Along with (3-1), the additional condition that ensures the survival of the true path is necessary for exact support identification, which is given as (3-2). In our analysis, both (3-1) and (3-2) are guaranteed under the results in Theorems 1 and 2, respectively, and these theorems jointly provide the overall sufficient condition for exact recovery in Theorem 3.

First, we obtain the sufficient condition for (3-1). Let *κ* be the largest correlation in magnitude between y and the columns associated with correct indices (j∈T) and *ζ* be the *K*-th largest correlation in magnitude between y and the columns corresponding to incorrect indices ($j\in T^{c}$). That is,

$$\kappa =\max_{j\in T}|a_{j}^{\prime }y|,\;\zeta =\min_{j\in I_{K}}\left|a_{j}^{\prime }y\right|$$

where $a_{j}$ is the *j*-th column of A and $I_{K}=\arg\max_{\left|I\right|=K,I\subset T^{c}}{∥A_{I}^{\prime }y∥}_{2}$. The following lemma provides the lower and the upper bounds of *κ* and *ζ*, respectively.

**Lemma** **5.** *κ and ζ satisfy:*

(8) $$\kappa \geq \frac{1-\delta_{2K}}{\sqrt{K}}{∥s_{T}∥}_{2},\;\zeta \leq \frac{\delta_{2K}}{\sqrt{K}}{∥s_{T}∥}_{2}.$$

**Proof of Lemma** **5.** See Appendix A. ☐

Using Lemma 5, one can obtain the sufficient condition for (3-1).

**Theorem 1** (Sufficient condition for (3-1))**.** *At least one support element is found in the pre-scanning stage under:*

(9) $$\delta_{2K}<0.5.$$

**Proof of Theorem** **1.** In order to choose at least one correct index in the pre-scanning stage, we should have κ>ζ. From Lemma 5, we can easily obtain the desired result.  ☐

Next, the condition (3-2) is guaranteed if the posterior indices of a true path always contain the support elements, that is $\left\{{\hat{t}}_{i+1},\;\cdots ,\;{\hat{t}}_{K}\right\}=\Omega \setminus \Lambda^{i}$ where $\Lambda^{i}\subset T$. This is because $∥r_{T}{∥}_{2}=0$, and thus, the condition (3-2) always holds for any positive pruning threshold *ϵ* as:

(10) $$∥r_{\Lambda^{i}\cup \left\{{\hat{t}}_{i+1},\;\cdots ,\;{\hat{t}}_{K}\right\}}{∥}_{2}={∥r_{T}∥}_{2}<\epsilon .$$

As mentioned, the problem to choose the posterior index set for a given true path $\Lambda^{i}\subset T$ is equivalent to the problem of reconstructing the (K−i)-sparse signal from the measurement $r_{\Lambda^{i}}$. Before we proceed, we provide useful definitions in our analysis. Let $\Upsilon_{l}$ be the combination of $\Lambda^{i}$ and $\left\{{\hat{t}}_{i+1},\;\cdots ,\;{\hat{t}}_{i+l}\right\}$ where 1≤l≤K−i ($\Upsilon_{l}=\Lambda^{i}\cup \left\{{\hat{t}}_{i+1},\;\cdots ,\;{\hat{t}}_{i+l}\right\}$). Next, let $\lambda^{l}$ be the largest correlation in magnitude between the residual $r_{\Upsilon_{l}}$ and columns associated with correct indices and $\gamma^{l}$ be the (K−i)-th largest correlation in magnitude between $r_{\Upsilon_{l}}$ and columns associated with incorrect indices. That is,

$$\lambda^{l}=\max_{j\in T\setminus \Upsilon_{l}}|a_{j}^{\prime }r_{\Upsilon_{l}}|,\;\gamma^{l}=\min_{j\in D_{i}}\left|a_{j}^{\prime }r_{\Upsilon_{l}}\right|$$

where $D_{i}=\arg\max_{|D|=K-i,D\subset \Omega \setminus T}{∥A_{D}^{\prime }r_{\Upsilon_{l}}∥}_{2}$. In the following lemma, we provide the lower bound of $\lambda^{l}$ and the upper bound of $\gamma^{l}$.

**Lemma** **6.** *If $\Upsilon_{l}\subset T$, then:*

(11) $$\lambda^{l}\geq \frac{1-\delta_{K}}{\sqrt{K-1}}∥{\bar{s}}_{T\cup \Upsilon_{l}}{∥}_{2},\;\gamma^{l}\leq \frac{\delta_{2K-1}}{\sqrt{K-1}}{∥{\bar{s}}_{T\cup \Upsilon_{l}}∥}_{2}.$$

*where ${\bar{s}}_{T\cup \Upsilon^{i}}={\left[s_{T\setminus \Upsilon^{i}}-{\left(A_{\Upsilon^{i}}^{\prime }A_{\Upsilon^{i}}\right)}^{-1}A_{\Upsilon^{i}}^{\prime }A_{T\setminus \Upsilon^{i}}s_{T\setminus \Upsilon^{i}}\right]}^{\prime }$.*

**Proof of Lemma** **6.** See Appendix B.  ☐

The following theorem provides the sufficient condition for (3-2).

**Theorem 2** (Sufficient condition for (3-2))**.** *The posterior index set of a true path $\Lambda^{i}\subset T$ consists only of correct ones under:*

(12) $$\delta_{2K-1}<0.5.$$

*for any 1≤i≤K−1.*

**Proof of Theorem** **2.** The element of posterior index set ${\hat{t}}_{i+l}$ becomes ${\hat{t}}_{i+l}\in T$ for any 1≤l≤K−i if the inequality $\lambda^{l}>\gamma^{l}$ is satisfied. That is, from Lemma 6, we have:

$$\delta_{K}+\delta_{2K-1}<1.$$

From Lemma 1, this inequality can be rewritten as $2\delta_{2K-1}<1$, which is the desired result.  ☐

The overall recovery condition of TPMP can be obtained by combining Theorems 1 and 2.

**Theorem 3** (Recovery condition of TPMP)**.** *TPMP exactly identifies the support of any K-sparse signal s from y=As under:*

(13) $$\begin{array}{c} \delta_{2K}<0.5. \end{array}$$

**Proof of Theorem** **3.** The condition (Equation (13)) is obtained by choosing a stricter condition between Theorems 1 and 2.  ☐

It is worthwhile to note that TPMP provides a more relaxed recovery condition than conventional greedy algorithms, such as OMP $\left(\delta_{K+1}<\frac{1}{\sqrt{K}+1}\right)$ [21], SP ($\delta_{3K}<0.165$) [23], gOMP $\left(\delta_{K^{2}}<\frac{\sqrt{L}}{\sqrt{L}+\sqrt{K}}\right)$ [35] and MMP ($\delta_{2K}<0.33$). In addition, our condition achieves the state-of-the-art recovery bound for the greedy algorithm, which was presented in [24].

### 3.2. Reconstruction from Noisy Measurements

We also consider reconstructing ECG when the compressed signal y is distorted by noise. In this scenario, the measurement y is defined as:

y=Φx+v=As+v

where v is an additive noise vector. Using the new expression of y containing v, we analyze the condition of TPMP to accurately identify the support by following the main architecture of the proofs for the noiseless scenario. Two requirements of TPMP to identify the support are (1) at least one support element should be chosen in the pre-scanning process (i.e., T∩Ω≠∅) (Theorem 4) and (2) true path ($\Lambda^{i}\subset T$) should survive the pruning strategy (Theorem 7). It is worth noting that while the pre-scanning condition (Theorem 4) is similar to that in the previous section, the search tree condition (Theorem 7) should satisfy the additional requirement compared to the result in Theorem 2. In the noiseless scenario (i.e., v=0), the support *T* always survives the pruning strategy whenever it is detected once since $∥r_{T}{∥}_{2}=0$ is the minimum residual in magnitude. On the other hand, in the presence of noise (i.e., v≠0), the additional guarantee of the support having the minimum residual in magnitude is required, that is,

$$\arg\min_{\Lambda_{K}}{∥r_{\Lambda_{K}}∥}_{2}=T$$

should hold to ensure the search tree condition.

Before we proceed, we provide a useful lemma in our analysis.

**Lemma** **7.** *For any $\Upsilon_{l}\subset T$, ${\bar{s}}_{T\cup \Upsilon_{l}}={\left[s_{T\setminus \Upsilon_{l}}-z_{\Upsilon_{l}}\right]}^{\prime }$ satisfies:*

(14) $$\begin{array}{c} ∥{\bar{s}}_{T\cup \Upsilon_{l}}{∥}_{2}\geq \frac{\sqrt{2(1+\delta_{K}^{2})}}{1+\delta_{K}}{∥s_{T\setminus \Upsilon_{l}}∥}_{2}. \end{array}$$

**Proof of Lemma** **7.** Since $z_{\Lambda^{i}}={\left(A_{\Lambda^{i}}^{\prime }A_{\Lambda^{i}}\right)}^{-1}A_{\Lambda^{i}}^{\prime }A_{T\setminus \Lambda^{i}}s_{T\setminus \Lambda^{i}}$ (see Lemma 2), $∥{\bar{s}}_{T\cup \Upsilon_{l}}{∥}_{2}^{2}$ is:

(15) $$\begin{array}{ccc} ∥{\bar{s}}_{T\cup \Upsilon_{l}}{∥}_{2}^{2} & = & {∥\left[\begin{array}{c} s_{T\setminus \Upsilon_{l}}\\ -{\left(A_{\Upsilon_{l}}^{\prime }A_{\Upsilon_{l}}\right)}^{-1}A_{\Upsilon_{l}}^{\prime }A_{T\setminus \Upsilon_{l}}s_{T\setminus \Upsilon_{l}} \end{array}\right]∥}_{2}^{2} \end{array}$$

(16) $$\begin{array}{ccc} & = & ∥s_{T\setminus \Upsilon_{l}}{∥}_{2}^{2}+{∥{\left(A_{\Upsilon_{l}}^{\prime }A_{\Upsilon_{l}}\right)}^{-1}A_{\Upsilon_{l}}^{\prime }A_{T\setminus \Upsilon_{l}}s_{T\setminus \Upsilon_{l}}∥}_{2}^{2}. \end{array}$$

From Lemma 2 and Definition 1, we then have:

(17) $$\begin{array}{c} ∥{\left(A_{\Upsilon_{l}}^{\prime }A_{\Upsilon_{l}}\right)}^{-1}A_{\Upsilon_{l}}^{\prime }A_{T\setminus \Upsilon_{l}}s_{T\setminus \Upsilon_{l}}{∥}_{2}^{2}\\ \;\geq \;\frac{1}{{\left(1+\delta_{|\Upsilon_{l}|}\right)}^{2}}{∥A_{\Upsilon_{l}}A_{T\setminus \Upsilon_{l}}s_{T\setminus \Upsilon_{l}}∥}_{2}^{2}\\ \;\geq \;\frac{1-\delta_{\Upsilon_{l}}}{{\left(1+\delta_{|\Upsilon_{l}|}\right)}^{2}}{∥A_{T\setminus \Upsilon_{l}}s_{T\setminus \Upsilon_{l}}∥}_{2}^{2}\\ \;\geq \;\frac{\left(1-\delta_{\Upsilon_{l}}\right)\left(1-\delta_{T\setminus \Upsilon_{l}}\right)}{{\left(1+\delta_{|\Upsilon_{l}|}\right)}^{2}}{∥A_{T\setminus \Upsilon_{l}}s_{T\setminus \Upsilon_{l}}∥}_{2}^{2}\\ \;\geq \;\frac{{\left(1-\delta_{K}\right)}^{2}}{{\left(1+\delta_{K}\right)}^{2}}{∥s_{T\setminus \Upsilon_{l}}∥}_{2}^{2}. \end{array}$$

From Equations (16) and (17), we obtain the lower bound of $∥{\bar{s}}_{T\cup \Upsilon_{l}}{∥}_{2}$ as:

(18) $$\begin{array}{ccc} ∥{\bar{s}}_{T\cup \Upsilon_{l}}{∥}_{2}^{2} & \geq & ∥s_{T\setminus \Upsilon_{l}}{∥}_{2}^{2}+\frac{{\left(1-\delta_{K}\right)}^{2}}{{\left(1+\delta_{K}\right)}^{2}}{∥s_{T\setminus \Upsilon_{l}}∥}_{2}^{2}\\ & \geq & \frac{2(1+\delta_{K}^{2})}{{\left(1+\delta_{K}\right)}^{2}}{∥s_{T\setminus \Lambda^{i}}∥}_{2}^{2} \end{array}$$

which is the desired result.  ☐

We first analyze the condition ensuring at least one support element is chosen by pre-scanning from noisy measurements. Let *ρ* be the largest correlation in magnitude between y, and the columns associated with correct indices and *η* be the *K*-th largest correlation in magnitude between y and the columns associated with incorrect indices. That is,

$$\begin{array}{c} \rho =\max_{j\in T}|a_{j}^{\prime }y|,\;\eta =\min_{j\in I_{K}}\left|a_{j}^{\prime }y\right| \end{array}$$

where $I_{K}=\arg\max_{\left|I\right|=K,I\subset T^{c}}{∥A_{I}^{\prime }y∥}_{2}$.

In the following lemmas, we provide the lower bound of *ρ* and the upper bound of *η*.

**Lemma** **8.** *ρ satisfies:*

(19) $$\begin{array}{c} \rho \geq \frac{1}{\sqrt{K}}\left[\left(1-\delta_{K}\right)∥s_{T}{∥}_{2}-\sqrt{1+\delta_{K}}{∥v∥}_{2}\right] \end{array}$$

*and η satisfies:*

(20) $$\begin{array}{c} \eta \leq \frac{1}{\sqrt{K}}\left[\delta_{2K}∥s_{T}{∥}_{2}+\sqrt{1+\delta_{K}}{∥v∥}_{2}\right]. \end{array}$$

**Proof of Lemma** **8.** See Appendix C.  ☐

The following theorem provides the condition ensuring that at least one support element is identified by the pre-scanning.

**Theorem 4** (Pre-scanning condition)**.** *At least one element in the support is found in the pre-scanning stage if the nonzero entries of the original sparse signal s satisfy:*

(21) $$\begin{array}{c} \min_{j\in T}|s_{j}|>\frac{2\sqrt{1+\delta_{K}}}{\sqrt{K}\left(1-\delta_{K}-\delta_{2K}\right)}{∥v∥}_{2}. \end{array}$$

**Proof of Theorem** **4.** It is clear that at least one support element (j∈T) is chosen in the pre-scanning if:

(22) $$\begin{array}{c} \rho >\eta . \end{array}$$

From Lemma 8, Equation (22) can be rewritten as:

(23) $$\begin{array}{c} \frac{1}{\sqrt{K}}\left[\left(1-\delta_{K}\right)∥s_{T}{∥}_{2}-\sqrt{1+\delta_{K}}{∥v∥}_{2}\right]>\frac{1}{\sqrt{K}}\left[\delta_{2K}∥s_{T}{∥}_{2}+\sqrt{1+\delta_{K}}{∥v∥}_{2}\right] \end{array}$$

and thus, we have:

(24) $$\begin{array}{c} ∥s_{T}{∥}_{2}>\frac{2\sqrt{1+\delta_{K}}}{1-\delta_{K}-\delta_{2K}}{∥v∥}_{2}. \end{array}$$

Since $∥s_{T}{∥}_{2}\geq \sqrt{K}\min_{j\in T}\left|s_{j}\right|$, we obtain the desired result as:

(25) $$\begin{array}{c} \min_{j\in T}|s_{j}|>\frac{2\sqrt{1+\delta_{K}}}{\sqrt{K}\left(1-\delta_{K}-\delta_{2K}\right)}{∥v∥}_{2}. \end{array}$$

☐

Next, we analyze the sufficient condition ensuring that the true path is not removed from the search tree. This requirement holds if (1) the posterior indices $\left\{{\hat{t}}_{i+1},\;\cdots ,\;{\hat{t}}_{K}\right\}$ of any true path $\Lambda^{i}\subset T$ satisfy $\left\{{\hat{t}}_{i+1},\;\cdots ,\;{\hat{t}}_{K}\right\}=T\setminus \Lambda^{i}$ and (2) the corresponding $\Lambda_{K}=\Lambda^{i}\cup \left\{{\hat{t}}_{i+1},\;\cdots ,\;{\hat{t}}_{K}\right\}=T$ satisfies $∥r_{\Lambda_{K}}{∥}_{2}<\epsilon$. For obtaining the condition ensuring $\left\{{\hat{t}}_{i+1},\;\cdots ,\;{\hat{t}}_{K}\right\}=T\setminus \Lambda^{i}$ for any $\Lambda^{i}\subset T$, let $\beta^{l}$ be the largest correlation in magnitude between correct indices and $r_{\Upsilon_{l}}$ and $\alpha^{l}$ be the (K−i)-th largest correlation in magnitude between $r_{\Upsilon_{l}}$ and columns associated with incorrect indices. That is,

$$\begin{array}{ccc} \beta^{l} & = & \arg\max_{j\in T\setminus \Upsilon_{l}}\left|a_{j}^{\prime }r_{\Upsilon_{l}}\right|,\\ \alpha^{l} & = & \arg\min_{j\in D_{i}}\left|a_{j}^{\prime }r_{\Upsilon_{l}}\right| \end{array}$$

where $D_{i}=\arg\max_{|D|=K-i,D\subset \Omega \setminus T}{∥A_{D}^{\prime }r_{\Upsilon_{l}}∥}_{2}$. The following lemma provides the lower bound of $\beta^{l}$ and the upper bound of $\alpha^{l}$.

**Lemma** **9.** *For any $\Upsilon_{l}\subset T$, $\beta^{l}$ and $\alpha^{l}$ satisfy:*

(26) $$\begin{array}{c} \beta^{l}>\frac{1}{\sqrt{K-1}}\left[\left(1-\delta_{K}\right)∥{\bar{s}}_{T\cup \Upsilon_{l}}{∥}_{2}-\sqrt{1+\delta_{K-1}}{∥v∥}_{2}\right] \end{array}$$

*and:*

(27) $$\begin{array}{c} \alpha^{l}<\frac{1}{\sqrt{K-1}}\left[\delta_{2K-1}∥{\bar{s}}_{T\cup \Upsilon_{l}}{∥}_{2}+\sqrt{1+\delta_{K-1}}{∥v∥}_{2}\right], \end{array}$$

*respectively.*

**Proof of Lemma** **9.** See Appendix D.  ☐

The guaranteed condition of the posterior indices to satisfy $\left\{{\hat{t}}_{i+1},\;\cdots ,\;{\hat{t}}_{K}\right\}=T\setminus \Lambda^{i}$ can be identified by combining Lemmas 7 and 9.

**Theorem** **5.** *For any $\Lambda^{i}\subset T$, the posterior indices satisfy $\left\{{\hat{t}}_{i+1},\;\cdots ,\;{\hat{t}}_{K}\right\}=T\setminus \Lambda^{i}$ under:*

(28) $$\begin{array}{c} \min_{j\in T}|s_{j}|>\frac{\left(1+\delta_{K}\right)\sqrt{2(1+\delta_{K-1})}}{\left(1-\delta_{K}-\delta_{2K-1}\right)\sqrt{1+\delta_{K}^{2}}}{∥v∥}_{2}. \end{array}$$

**Proof of Theorem** **5.** Similar to Theorem 2, one can notice that the posterior indices of the true path $\Lambda^{i}$ contain only true indices for any 1≤l≤K−i if:

(29) $$\beta^{l}>\alpha^{l},$$

which can be rewritten by using Lemma 9 as:

(30) $$\begin{array}{c} \frac{1}{\sqrt{K-1}}\left[\left(1-\delta_{K}\right)∥{\bar{s}}_{T\cup \Upsilon }{∥}_{2}-\sqrt{1+\delta_{K-1}}{∥v∥}_{2}\right]>\frac{1}{\sqrt{K-1}}\left[\delta_{2K-1}∥{\bar{s}}_{T\cup \Upsilon_{l}}{∥}_{2}+\sqrt{1+\delta_{K-1}}{∥v∥}_{2}\right] \end{array}$$

where $\Upsilon_{l}\subset T$. After some manipulations, we have:

(31) $$\begin{array}{c} ∥{\bar{s}}_{T\cup \Upsilon_{l}}{∥}_{2}>\frac{2\sqrt{1+\delta_{K-1}}}{1-\delta_{K}-\delta_{2K-1}}{∥v∥}_{2}. \end{array}$$

Recall Equation (14) from Lemma 7 that:

$$∥{\bar{s}}_{T\cup \Upsilon_{l}}{∥}_{2}\geq \frac{\sqrt{2(1+\delta_{K}^{2})}}{1+\delta_{K}}{∥s_{T\setminus \Upsilon_{l}}∥}_{2}$$

and since $∥s_{T\cup \Upsilon_{l}}{∥}_{2}\geq \min_{j\in T}\left|s_{j}\right|$, we get the desired result.  ☐

Next, we provide the guaranteed condition under which the residual of the support satisfies:

(32) $$∥r_{T}{∥}_{2}\leq \epsilon$$

for any positive pruning threshold *ϵ*. Recall that the pruning threshold is updated by the smallest residual in magnitude among all $\Lambda_{K}$ found in each layer of the tree. Therefore, as long as Equation (32) holds and $\Lambda_{K}=T$ is found at least once, *T* has a smaller residual in magnitude than any possible pruning threshold *ϵ* and, thus, cannot be removed from the search tree.

**Theorem** **6.** *The support has the minimum residual in magnitude among all possible $\Lambda_{K}$ ($|\Lambda_{K}|=K$) if:*

(33) $$\min_{j\in T}\left|s_{j}\right|>\frac{\sqrt{2}\left(1+\delta_{K}\right){∥v∥}_{2}}{\sqrt{\left(1-\delta_{2K}\right)\left(1+\delta_{K}^{2}\right)}}.$$

**Proof of Theorem** **6.** See Appendix E.  ☐

If Theorems 5 and 6 jointly hold, then the condition that the true path $\Lambda^{i}$ is not removed can be guaranteed as follows.

**Theorem 7** (Search tree condition)**.** *The true path $\Lambda^{i}\subset T$ survives the pruning strategy for any i under:*

(34) $$\begin{array}{c} \min_{j\in T}|s_{j}{|>\max\left(\mu ,\omega \right)∥v∥}_{2} \end{array}$$

*where $\mu =\frac{\left(1+\delta_{K}\right)\sqrt{2(1+\delta_{K-1})}}{\left(1-\delta_{K}-\delta_{2K-1}\right)\sqrt{1+\delta_{K}^{2}}}$ and $\omega =\frac{\sqrt{2}\left(1+\delta_{K}\right)}{\sqrt{\left(1-\delta_{2K}\right)\left(1+\delta_{K}^{2}\right)}}$.*

**Proof of Theorem** **7.** Immediate from Theorems 5 and 6.  ☐

By combining the results from Theorems 4 and 7, we obtain the sufficient condition of exact support identification from noisy measurements.

**Theorem 8** (Exact support identification of TPMP)**.** *The TPMP algorithm accurately identifies the support from the noisy measurement y=As+v under:*

(35) $$\begin{array}{c} \min_{j\in T}|s_{j}{|>\max\left(\nu ,\mu ,\omega \right)∥v∥}_{2}=\gamma {∥v∥}_{2} \end{array}$$

*where $\nu =\frac{2\sqrt{1+\delta_{K}}}{\sqrt{K}\left(1-\delta_{K}-\delta_{2K}\right)}$, $\mu =\frac{\left(1+\delta_{K}\right)\sqrt{2(1+\delta_{K-1})}}{\left(1-\delta_{K}-\delta_{2K-1}\right)\sqrt{1+\delta_{K}^{2}}}$ and $\omega =\frac{\sqrt{2}\left(1+\delta_{K}\right)}{\sqrt{\left(1-\delta_{2K}\right)\left(1+\delta_{K}^{2}\right)}}$.*

**Proof of Theorem** **8.** Immediate from Theorems 4 and 7.  ☐

Note that the sufficient condition given in Equation (35) infers that the signal-to-noise ratio (SNR) of the sparse signal should be higher than the constant *γ*. If Equation (35) holds, the support *T* can be exactly identified, and the signal reconstruction is based on the columns of A associated with *T*. In this sense, the system is equivalent to the overdetermined system ($y=A_{T}s_{T}+v$) and achieves identical performance to the best possible estimator referred to as the oracle estimator ${\hat{s}}_{T}=A_{T}^{†}y$.

## 4. Simulation and Discussion

**Figure 4:**
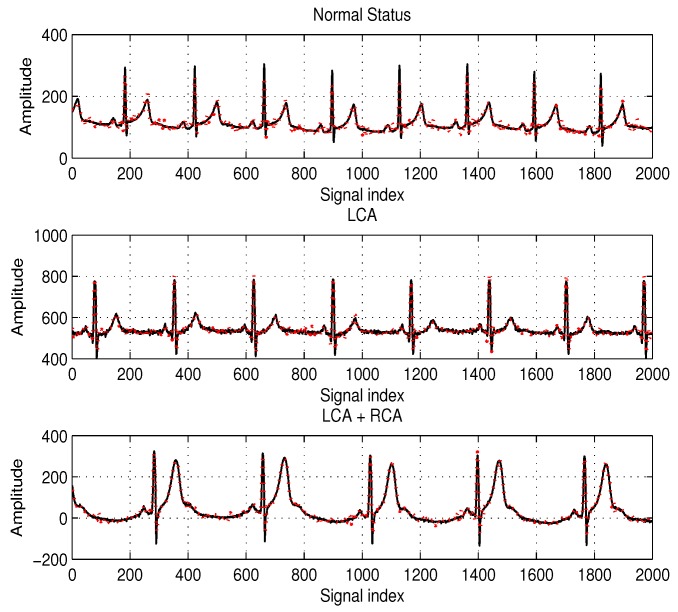
Examples of the tested ECG samples in our simulations and the reconstructed signal for each sample signal by tree pruning-based matching pursuit (TPMP) at compression ratio (CR) =65, where the solid and dotted lines denote the original ECG (x) and the reconstructed ECG ($\hat{x}=\Psi \hat{s}$), respectively.

In this section, we evaluate the numerical ECG recovery performance of the proposed TPMP algorithm and the existing sparse recovery algorithms. The simulation is based on the discrete cosine transform (DCT) basis matrix Ψ∈N×N, the random Bernoulli sensing matrix Φ∈M×N (M≪N) where each element of **Φ** is either $\pm \frac{1}{\sqrt{M}}$, and the measurement is distorted by an additive noise vector (according to the results in [19], we set the signal-to-noise ratio (SNR) as 40 dB when v≠0) $v∼N(0,\sigma^{2}I)$. In the simulation, we check the reconstruction performance by performing at least 5000 independent trials for each number of measurements *M*, which is directly related to the compression ratio (CR) defined as CR $=\frac{N-M}{N}\times 100$ (%). In addition, we used two measures for the performance evaluation: (1) the exact recovery ratio (ERR), which is the probability of the exact identification of the support of s ($T=\{j\;|\;s_{j}\neq 0\}$) and (2) the percentage root-mean-square difference (PRD), which is defined as:

$$PRD=\frac{∥\tilde{x}-\hat{x}{∥}_{2}}{∥\tilde{x}-E\left[\tilde{x}\right]{∥}_{2}}$$

where $\tilde{x}$ is the digitized signal of original ECG and $\hat{x}$ is the reconstructed ECG. We exploited six ECG samples from the European ST-TDatabase in Physionet [36], and a randomly chosen window of 1000 continuative signal samples among all the data is used for each trial with sparsity level K=100. The samples are measured from distinct patients including people with normal status, left circumflex artery (LCA) or right coronary artery (RCA) diseases (see Figure 4). Each record is two hours in duration and contains two signals, each sampled at 250 samples per second with 12-bit resolution over a nominal 20-mV input range. The sample values were rescaled after digitization with reference to calibration signals in the original analog recordings, in order to obtain a uniform scale of 200 ADC units per mV for all signals, and each of the signal files is 5,400,000 bytes long. All algorithms under test are coded by MATLAB software and run by a personal computer with an Intel Core i5 processor and Microsoft Windows 7.

- Oracle estimator [32]
- Linear MMSE
- Basis pursuit (BP) [9]
- Orthogonal matching pursuit (OMP) [21]
- Subspace pursuit (SP) [23]
- Multipath matching pursuit (MMP) [19] with L=2
- Depth-first multipath matching pursuit (MMP-DF) [19] with L=2: MMP with reduced complexity
- TPMP (c=0, 1)

**Figure 5:**
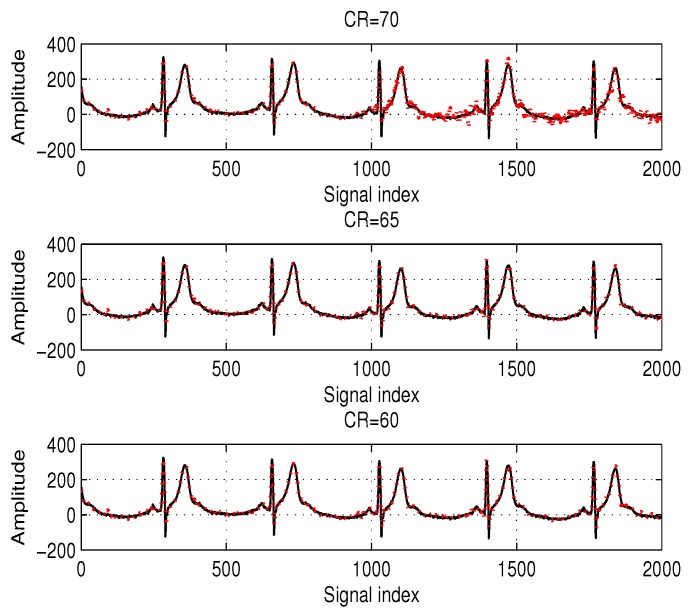
ECG reconstruction using the proposed method when N=1000 and K=100 where the solid and dotted lines denote the original ECG (x) and the reconstructed ECG ($\hat{x}=\Psi \hat{s}$), respectively.

Before the discussion, it is clear that higher CR (or smaller *M*) degrades the reconstruction performance (see Figure 5), and thus, we demonstrate the effectiveness of the proposed method by showing that TPMP requires minimum *M* for accurately identifying the support.

**Figure 6:**
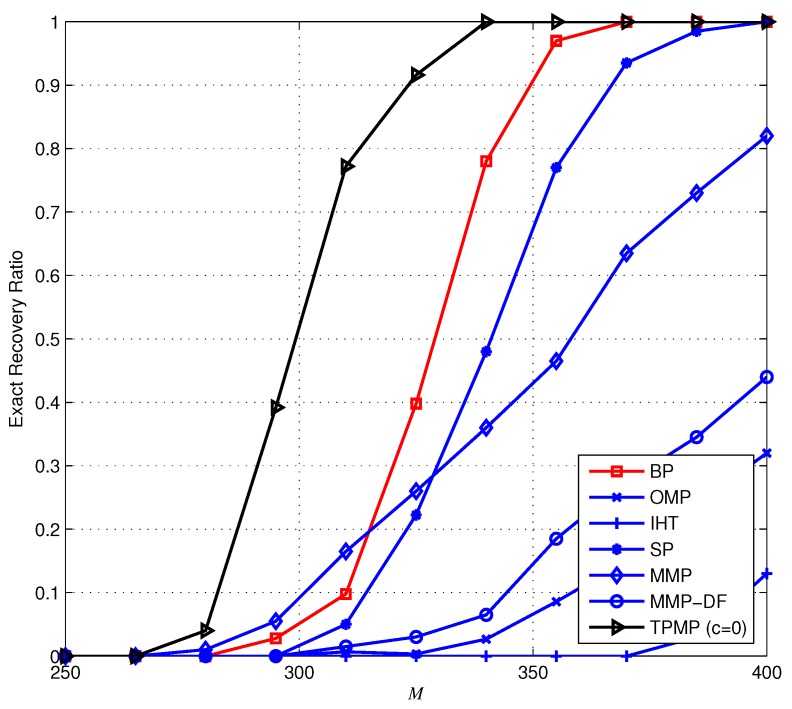
Exact recovery ratio (ERR) performance of the sparse signal recovery methods when N=1000 and K=100.

**Figure 7:**
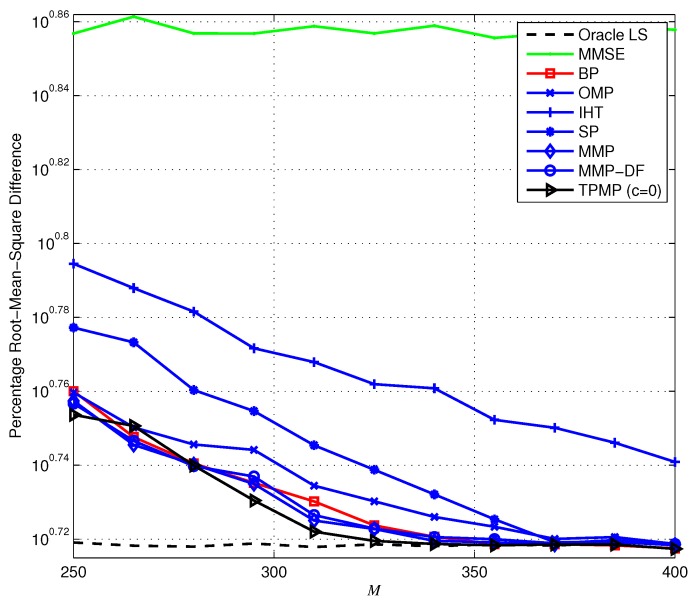
Percentage root-mean-square difference (PRD) performance of the sparse signal recovery methods when N=1000 and K=100.

**Figure 8:**
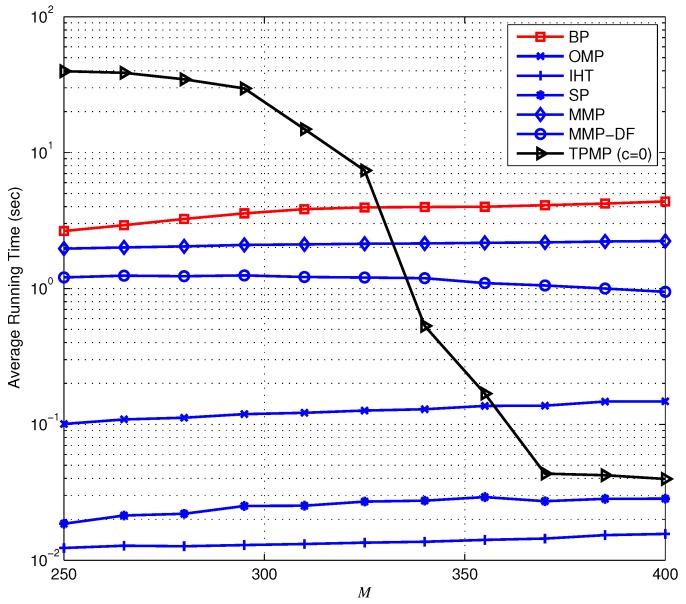
Complexity of the sparse signal recovery methods when N=1000 and K=100.

First, we evaluate the ECG reconstruction performance from noiseless measurements (y=Φx). Figure 6 provides the ERR performance when K=100 (i.e., $\frac{K}{N}=10\%$) as a function of *M*. Overall, we observe that TPMP performs better than existing algorithms, in particular for small *M*. In particular, it is shown that while ERR of TPMP drops moderately with the decrement of *M*, that of other conventional algorithms drops sharply and fails to provide reliable recovery performance. In Figure 7, we plot the PRD performance of the sparse recovery methods. Note that since the exact support information is given to the oracle estimator, it can be regarded to have the lower bound of PRD (since $\tilde{x}$ is approximated to x, PRD determined by the approximation error $∥\tilde{x}{-x∥}_{2}$ is the lower bound of PRD). Due to the multiple candidate investigation, we observe that TPMP reaches the optimal performance with minimum *M* among tested algorithms. For instance, while SP requires at least M=385 measurements for optimal performance, TPMP requires only M=325. In addition, while MMP provides lower PRD for very small *M*, the PRD of TPMP reaches the best possible performance with smaller *M*. This demonstrates that TPMP not only outperforms conventional methods in reconstruction accuracy, but also enables reduction in data storage. Figure 8 shows the running time complexity. Since TPMP performs the tree search, it is no wonder that the running time complexity of TPMP can be large. Interestingly, we observe that the complexity of TPMP becomes similar to that of conventional greedy algorithms in the large *M* regime. This is because $∥r_{T}{∥}_{2}=0$, and thus, whenever any path satisfying $∥r_{\Lambda_{K}}{∥}_{2}=0$ is found, we regard $\Lambda_{K}$ to be the support and immediately stop the search. In this sense, the support can be identified in the early layer of the search.

**Figure 9:**
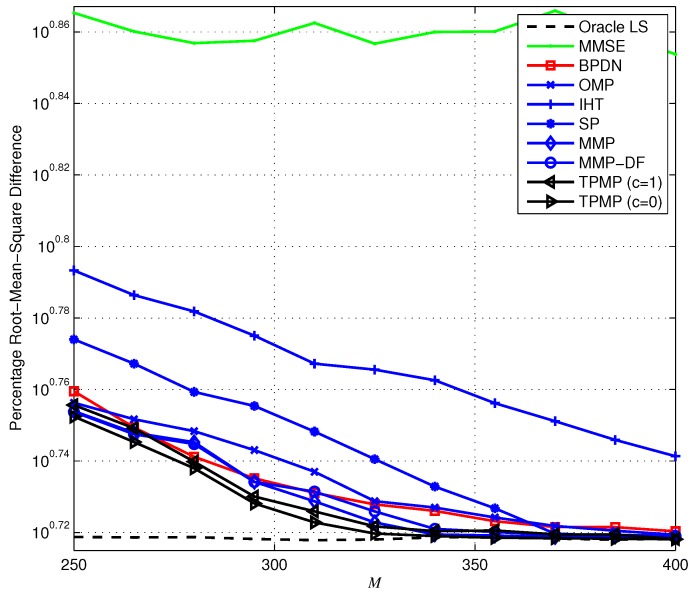
PRD performance of the sparse signal reconstruction from noisy measurements.

**Figure 10:**
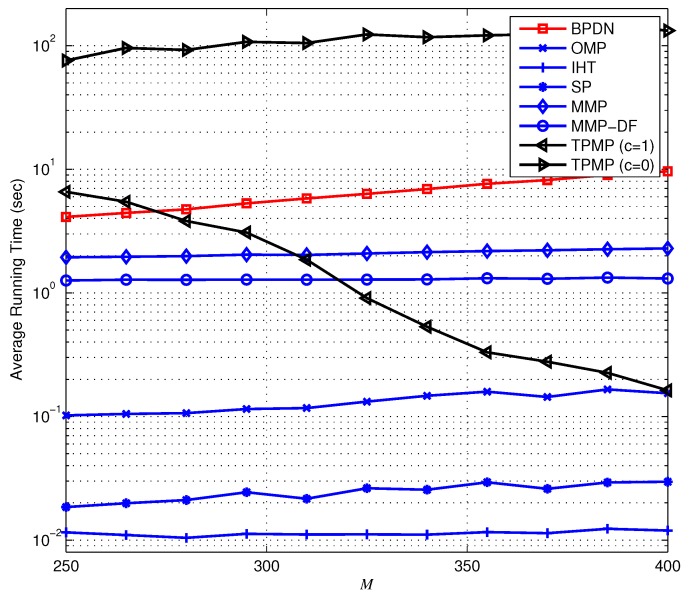
Average running time complexity of the sparse signal reconstruction from noisy measurements.

Next, we provide the recovery performance in the presence of noise, that is when the measurement is defined as y=Φx+v. Recall that in the noiseless scenario, the search could be finished in the early stage whenever any $\Lambda_{K}$ satisfying $∥r_{\Lambda_{K}}{∥}_{2}=0$ is found with c=0. However, this is no longer valid in the presence of noise since $∥r_{T}{∥}_{2}={∥P_{T}^{⊥}v∥}_{2}\neq 0$, and thus, we assume positive *c* in the noisy setting. Note that this stopping criterion does not affect the recovery condition since if Theorem 8 holds, then $∥r_{T}{∥}_{2}$ still has the minimum residual in magnitude, and thus, *T* is selected as the support whether $∥r_{T}{∥}_{2}^{2}>cN\sigma^{2}$ or not. Therefore, one can notice that *c* is used only to shut down the search earlier than the original TPMP, and thus proper choice of *c* is required for the minimum tradeoff between the numerical performance and the complexity. In Figure 9, we plot the PRD performance of the sparse recovery algorithms. Similar to the noiseless scenario, we observe that the proposed TPMP algorithm outperforms conventional methods. In particular, the PRDs of TPMP with both c=0 and c=1 are smaller than that of MMP. To be specific, we observe in Figure 9 that TPMP performs closest to the lower bound of PRD (PRD of oracle LS) among all of the tested algorithms. In order to demonstrate the validity of real-time implementation, Figure 10 provides the average running time complexity of the sparse recovery algorithms as a function of *M*. Similar to the results in Figure 8, the running time complexity of TPMP is the highest due to the tree search, especially for small *M*. Nevertheless, the computational burden of TPMP can be substantially reduced by limiting the minimum pruning threshold determined by *c*. In particular, if c=1, significant complexity reduction over the original TPMP (c=0) is achieved, and it performs with similar complexity as OMP. In addition, since TPMP with c=1 performs similar to MMP with smaller complexity than MMP and MMP-DF, one can notice that TPMP provides a better tradeoff between the performance and the complexity than MMP.

## 5. Conclusions

In this work, we proposed an effective ECG reconstruction method referred to as tree pruning-based matching pursuit (TPMP). In order to improve the accuracy of ECG recovery for large CR (or small *M*), the TPMP algorithm performs the tree search and investigates multiple promising candidates. Further, the complexity overhead caused by the tree search is reduced by the pruning strategy. In our analysis, we analyzed the sufficient condition of TPMP for exactly identifying the support, which provides an improved recovery bound compared to the existing methods. In addition, our numerical results discussed in this work demonstrate that TPMP provides improved performance with competitive complexity with conventional algorithms.

## Figures and Tables

**Table 1:** The TPMP algorithm.

**Input:** Measurement **y**, sensing matrix **A**, sparsity *K*, 0<c<1	
**Output:** Reconstructed ECG $\hat{x}$	
**Initialization:** i:=0, $W^{0}:=\emptyset$, ϵ=∞	
$\Theta =\arg\max_{|I_{K}|=K}{∥A_{I_{K}}^{\prime }y∥}_{2}$	(*pre-scanning*)
**while** i<K **do**	
i:=i+1, $W^{i}:=\emptyset$, $\epsilon_{i+1}:=\epsilon_{i}$	
**for** l=1 **to** $|W^{i-1}|$ **do**	
$\theta :=\Theta \setminus \Lambda^{i-1}\left(l\right)$	
**for** j=1 **to** |θ| **do**	
$\Lambda^{i}:=\Lambda^{i-1}\left(l\right)\cup \left\{t_{i}\left(j\right)\right\}$	(*update j-th path*)
**if** $\Lambda^{i}\notin W^{i}$ **then**	(*check the duplicated path*)
Obtain $\left\{{\hat{t}}_{i+1},\;\cdots ,\;{\hat{t}}_{K}\right\}$	(*posterior index set construction*)
$\Lambda_{K}=\Lambda^{i}\cup \left\{{\hat{t}}_{i+1},\;\cdots ,\;{\hat{t}}_{K}\right\}$	
$r_{\Lambda_{K}}=P_{\Lambda_{K}}^{⊥}y$	
**if** $∥r_{\Lambda_{K}}{∥}_{2}\leq \epsilon_{i}$ **then**	(*pruning decision*)
$W^{i}:=W^{i}\cup \Lambda^{i}$, $I^{*}:=\Lambda_{K}$	
**if** $∥r_{I^{*}}{∥}_{2}^{2}\leq cN\sigma^{2}$ **then**	(*search termination*)
j=|θ|+1, $l=|W^{i-1}|+1$, i=K+1
**end if**	
**if** $∥r_{I^{*}}{∥}_{2}\leq \epsilon_{i+1}$ **then**	
$\epsilon_{i+1}:={∥r_{I^{*}}∥}_{2}$	(*update pruning threshold*)
**end if**	
**end if**	
**end if**	
**end for**	
**end for**	
**end while**	
${\hat{s}}^{*}=A_{I^{*}}^{†}y$	
**return** ${\hat{x}}^{*}=\Psi {\hat{s}}^{*}$	(*ECG reconstruction*)

## Appendix A. Proof of Lemma 5

From the definition of *κ*, we have:

(A1) $$\begin{array}{ccc} \kappa & = & \max_{j\in T}\left|a_{j}^{\prime }y\right|\\ & = & ∥A_{T}^{\prime }{y∥}_{\infty }\geq \frac{1}{\sqrt{K}}{∥A_{T}^{\prime }y∥}_{2} \end{array}$$

where (A1) is from the inequality ${∥u∥}_{\infty }\geq \frac{1}{\sqrt{{∥u∥}_{0}}}{∥u∥}_{2}$ for any vector u. Using Definition 1, we further have:

(A2) $$\begin{array}{ccc} \kappa & \geq & \frac{1}{\sqrt{K}}∥A_{T}^{\prime }{y∥}_{2}=\frac{1}{\sqrt{K}}{∥A_{T}^{\prime }As∥}_{2}\\ & = & \frac{1}{\sqrt{K}}{∥A_{T}^{\prime }A_{T}s_{T}∥}_{2}\\ & \geq & \frac{1-\delta_{K}}{\sqrt{K}}{∥s_{T}∥}_{2}\\ & > & \frac{1-\delta_{2K}}{\sqrt{K}}{∥s_{T}∥}_{2} \end{array}$$

where (A2) is from Lemma 2 and (A2) is from Lemma 1.

Next, from the definition of *ζ*, we have:

(A3) $$\begin{array}{c} \sqrt{K}\zeta \leq \sqrt{\sum_{j\in I_{K}}{\left|a_{j}^{\prime }y\right|}^{2}}={∥A_{I_{K}}^{\prime }y∥}_{2} \end{array}$$

where $I_{K}=\arg\max_{\left|I\right|=K,I\subset T^{c}}{∥A_{I}^{\prime }y∥}_{2}$. Since $I_{K}$ and *T* are disjoint ($I_{K}\subset T^{c}$) and $|I_{K}|=K$, we have:

(A4) $$\begin{array}{c} ∥A_{I_{K}}^{\prime }{y∥}_{2}=∥A_{I_{K}}^{\prime }A_{T}s_{T}{∥}_{2}\leq \delta_{2K}{∥s_{T}∥}_{2} \end{array}$$

where (A4) is from Lemma 3. From (A3) and (A4), we get the desired result.

## Appendix B. Proof of Lemma 6

For any $\Upsilon_{l}=\Lambda^{i}\cup \left\{{\hat{t}}_{i+1},\;\cdots ,\;{\hat{t}}_{i+l}\right\}\subset T$ and $\lambda^{l}=\max_{j\in T\setminus \Upsilon_{l}}\left|a_{j}^{\prime }r_{\Upsilon_{l}}\right|$, we have:

(B1) $$\begin{array}{ccc} \max_{j\in T\setminus \Upsilon_{l}}\left|a_{j}^{\prime }r_{\Upsilon_{l}}\right| & = & ∥A_{T\setminus \Upsilon_{l}}^{\prime }r_{\Upsilon_{l}}{∥}_{\infty }\\ & \geq & \frac{1}{\sqrt{|T\setminus \Upsilon_{l}|}}{∥A_{T\setminus \Upsilon_{l}}^{\prime }r_{\Upsilon_{l}}∥}_{2}\\ & = & \frac{1}{\sqrt{K-i-l}}{∥A_{T}^{\prime }A_{T\cup \Upsilon_{l}}{\bar{s}}_{T\cup \Upsilon_{l}}∥}_{2} \end{array}$$

where $P_{\Upsilon_{l}}^{⊥}=I-A_{\lambda^{l}}{\left(A_{\Upsilon_{l}}^{\prime }A_{\Upsilon_{l}}\right)}^{-1}A_{\Upsilon_{l}}^{\prime }$, and (B1) is from Definition 2. Since $T\cup \Psi_{l}=T$ ($\Psi_{l}\subset T$), (B1) can be rewritten from Lemma 2 as:

(B2) $$\begin{array}{ccc} \max_{j\in T\setminus \Upsilon_{l}}\left|a_{j}^{\prime }r_{\Upsilon_{l}}\right| & \geq & \frac{1}{\sqrt{K-i-l}}{∥A_{T}^{\prime }A_{T\cup \Upsilon_{l}}{\bar{s}}_{T\cup \Upsilon_{l}}∥}_{2}\\ & = & \frac{1}{\sqrt{K-i-l}}{∥A_{T}^{\prime }A_{T}{\bar{s}}_{T\cup \Upsilon_{l}}∥}_{2}\\ & \geq & \frac{1-\delta_{\left|T\right|}}{\sqrt{K-i-l}}{∥{\bar{s}}_{T\cup \Upsilon_{l}}∥}_{2}\\ & \geq & \frac{1-\delta_{\left|T\right|}}{\sqrt{K-i}}{∥{\bar{s}}_{T\cup \Upsilon_{l}}∥}_{2}\\ & > & \frac{1-\delta_{K}}{\sqrt{K}}{∥{\bar{s}}_{T\cup \Upsilon_{l}}∥}_{2} \end{array}$$

where (B2) is from Lemma 1.

Next, From the definition of $\gamma^{l}=\min_{j\in D_{K}}\left|a_{j}^{\prime }r_{\Upsilon_{l}}\right|$, we have:

(B3) $$\begin{array}{c} \sqrt{|D_{K}|}\gamma^{l}\leq \sqrt{\sum_{j\in D_{K}}{\left|a_{j}^{\prime }r_{\Upsilon_{l}}\right|}^{2}}={∥A_{D_{K}}^{\prime }r_{\Upsilon_{l}}∥}_{2} \end{array}$$

where $D_{K}=\arg\max_{|D|=K-i,D\subset \Omega \setminus T}{∥A_{D}^{\prime }r_{\Upsilon_{l}}∥}_{2}$ and $\Upsilon_{l}\subset T$. Using the triangle inequality, we have:

(B4) $$\begin{array}{ccc} ∥A_{D_{K}}^{\prime }r_{\Upsilon_{l}}{∥}_{2} & = & ∥A_{D_{K}}^{\prime }A_{T\cup \Upsilon_{l}}{\bar{s}}_{T\cup \Upsilon_{l}}{∥}_{2} \end{array}$$

(B5) $$\begin{array}{lll} & = & ∥A_{D_{K}}^{\prime }A_{T}{\bar{s}}_{T\cup \Upsilon_{l}}{∥}_{2} \end{array}$$

(B6) $$\begin{array}{lll} & \leq & \delta_{2K-i}{∥{\bar{s}}_{T\cup \Upsilon_{l}}∥}_{2} \end{array}$$

(B7) $$\begin{array}{lll} & < & \delta_{2K}{∥{\bar{s}}_{T\cup \Upsilon_{l}}∥}_{2} \end{array}$$

where (B4) is from Definition 2, (B5) is because $T\cup \Upsilon_{l}=T$, (B6) is from Lemma 3 and (B7) is from Lemma 1.

## Appendix C. Proof of Lemma 8

From the definition of *ρ*, we have:

(C1) $$\begin{array}{ccc} \rho & = & \max_{j\in T}|a_{j}^{\prime }y|=∥A_{T}^{\prime }{y∥}_{\infty }\\ & \geq & \frac{1}{\sqrt{\left|T\right|}}{∥A_{T}^{\prime }y∥}_{2}\\ & = & \frac{1}{\sqrt{K}}{∥A_{T}^{\prime }\left(A_{T}s_{T}+v\right)∥}_{2}\\ & \geq & \frac{1}{\sqrt{K}}\left(∥A_{T}^{\prime }A_{T}s_{T}{∥}_{2}-{∥A_{T}^{\prime }v∥}_{2}\right). \end{array}$$

From Lemma 2 and Definition 1, we have:

(C2) $$\begin{array}{c} ∥A_{T}^{\prime }A_{T}s_{T}{∥}_{2}\geq \left(1-\delta_{K}\right){∥s_{T}∥}_{2} \end{array}$$

and:

(C3) $$\begin{array}{c} ∥A_{T}^{\prime }{v∥}_{2}\leq \sqrt{1+\delta_{K}}{∥v∥}_{2}, \end{array}$$

respectively. Using (C2) and (C3), *ρ* is lower bounded as:

(C4) $$\begin{array}{c} \rho \geq \frac{1}{\sqrt{K}}\left[\left(1-\delta_{K}\right)∥s_{T}{∥}_{2}-\sqrt{1+\delta_{K}}{∥v∥}_{2}\right], \end{array}$$

which is the desired result.

From the definition of *η*, we have:

(C5) $$\begin{array}{c} \sqrt{K}\eta \leq \sqrt{\sum_{j\in I_{K}}{\left|a_{j}^{\prime }y\right|}^{2}}={∥A_{I_{K}}^{\prime }y∥}_{2} \end{array}$$

where $I_{K}=\arg\max_{\left|I\right|=K,I\subset T^{c}}{∥A_{I}^{\prime }y∥}_{2}$. Using the triangle inequality, we have:

(C6) $$\begin{array}{ccc} ∥A_{I_{K}}^{\prime }{y∥}_{2} & = & ∥A_{I_{K}}^{\prime }\left(A_{T}s_{T}+v\right){∥}_{2}\\ & \leq & ∥A_{I_{K}}^{\prime }A_{T}s_{T}{∥}_{2}+{∥A_{I_{K}}^{\prime }v∥}_{2}. \end{array}$$

Since $I_{K}$ and *T* are disjoint ($I_{K}\subset T^{c}$), we further have:

(C7) $$\begin{array}{c} ∥A_{I_{K}}^{\prime }A_{T}s_{T}{∥}_{2}\leq \delta_{2K}{∥s_{T}∥}_{2} \end{array}$$

from Lemma 3 and:

(C8) $$\begin{array}{ccc} ∥A_{I_{K}}^{\prime }{v∥}_{2} & \leq & \sqrt{1+\delta_{K}}{∥v∥}_{2} \end{array}$$

from Definition 1. Using (C7) and (C8), we have:

(C9) $$\begin{array}{c} ∥A_{I_{K}}^{\prime }{y∥}_{2}\leq \delta_{2K}∥s_{T}{∥}_{2}+\sqrt{1+\delta_{K}}{∥v∥}_{2} \end{array}$$

and since $∥A_{I_{K}}^{\prime }{y∥}_{2}\geq \sqrt{K}\eta$, we have:

(C10) $$\begin{array}{c} \eta \leq \frac{1}{\sqrt{K}}\left[\delta_{2K}∥s_{T}{∥}_{2}+\sqrt{1+\delta_{K}}{∥v∥}_{2}\right], \end{array}$$

which is the desired result.

## Appendix D. Proof of Lemma 9

Since $\Upsilon_{l}\subset T$ and $\beta^{l}=\max_{j\in T\setminus \Upsilon_{l}}\left|a_{j}^{\prime }r_{\Upsilon_{l}}\right|$, we have:

(D1) $$\begin{array}{ccc} \max_{j\in T\setminus \Upsilon_{l}}\left|a_{j}^{\prime }r_{\Upsilon_{l}}\right| & = & ∥A_{T\setminus \Upsilon_{l}}^{\prime }r_{\Upsilon_{l}}{∥}_{\infty }\\ & \geq & \frac{1}{\sqrt{|T\setminus \Upsilon_{l}|}}{∥A_{T\setminus \Upsilon_{l}}^{\prime }P_{\Upsilon_{l}}^{⊥}y∥}_{2}\\ & = & \frac{1}{\sqrt{|T\setminus \Upsilon_{l}|}}{∥A_{T\setminus \Upsilon_{l}}^{\prime }P_{\Upsilon_{l}}^{⊥}\left(A_{T}s_{T}+v\right)∥}_{2}\\ & = & \frac{1}{\sqrt{K-i-l}}{∥A_{T}^{\prime }P_{\Upsilon_{l}}^{⊥}A_{T\setminus \Upsilon_{l}}s_{T\setminus \Upsilon_{l}}+A_{T\setminus \Upsilon_{l}}^{\prime }P_{\Upsilon_{l}}^{⊥}v∥}_{2}\\ & \geq & \frac{1}{\sqrt{K-i-l}}[∥A_{T}^{\prime }P_{\Upsilon_{l}}^{⊥}A_{T\setminus \Upsilon_{l}}s_{T\setminus \Upsilon_{l}}{∥}_{2}-∥A_{T\setminus \Upsilon_{l}}^{\prime }P_{\Upsilon_{l}}^{⊥}v{∥}_{2}]\\ & \geq & \frac{1}{\sqrt{K-i-l}}[∥A_{T}^{\prime }A_{T}{\bar{s}}_{T\cup \Upsilon_{l}}{∥}_{2}-∥A_{T\setminus \Upsilon_{l}}^{\prime }P_{\Upsilon_{l}}^{⊥}v{∥}_{2}] \end{array}$$

where (D2) is from Definition 2. From (B2) in Appendix B and Lemma 4, we have:

(D2) $$\begin{array}{c} ∥A_{T}^{\prime }A_{T}{\bar{s}}_{T\cup \Upsilon_{l}}{∥}_{2}>\left(1-\delta_{K}\right){∥{\bar{s}}_{T\cup \Upsilon_{l}}∥}_{2} \end{array}$$

and:

(D3) $$\begin{array}{ccc} ∥A_{T\setminus \Upsilon_{l}}^{\prime }P_{\Upsilon_{l}}^{⊥}{v∥}_{2} & \leq & ∥A_{T\setminus \Upsilon_{l}}^{\prime }{∥}_{2}\;{∥P_{\Upsilon_{l}}^{⊥}v∥}_{2}\\ & \leq & \sqrt{1+\delta_{|T\setminus \Upsilon_{l}|}}{∥P_{\Upsilon_{l}}^{⊥}v∥}_{2}\\ & \leq & \sqrt{1+\delta_{|T\setminus \Upsilon_{l}|}}{∥v∥}_{2}\\ & \leq & \sqrt{1+\delta_{K-i-l}}{∥v∥}_{2}, \end{array}$$

respectively. From (D2), (D2) and (D4), we have:

(D4) $$\begin{array}{ccc} \beta^{l} & > & \frac{1}{\sqrt{K-i-l}}\left[∥A_{T}^{\prime }A_{T}{\bar{s}}_{T\cup \Upsilon_{l}}{∥}_{2}-{∥A_{T\setminus \Upsilon_{l}}^{\prime }P_{\Upsilon_{l}}^{⊥}v∥}_{2}\right]\\ & > & \frac{1}{\sqrt{K-i-l}}\left[\left(1-\delta_{K}\right)∥{\bar{s}}_{T\cup \Upsilon_{l}}{∥}_{2}-\sqrt{1+\delta_{K-i-l}}{∥v∥}_{2}\right]\\ & > & \frac{1}{\sqrt{K-1}}\left[\left(1-\delta_{K}\right)∥{\bar{s}}_{T\cup \Upsilon }{∥}_{2}-\sqrt{1+\delta_{K-1}}{∥v∥}_{2}\right]. \end{array}$$

Next, from the definition of $\alpha^{l}=\min_{j\in D_{K}}\left|a_{j}^{\prime }r_{\Upsilon_{l}}\right|$, we have:

(D5) $$\begin{array}{c} \sqrt{|D_{K}|}\alpha^{l}\leq \sqrt{\sum_{j\in D_{K}}{\left|a_{j}^{\prime }r_{\Upsilon_{l}}\right|}^{2}}={∥A_{D_{K}}^{\prime }r_{\Upsilon_{l}}∥}_{2} \end{array}$$

where $D_{K}=\arg\max_{\left|D\right|=K-i,D\subset \Omega \setminus T^{\prime }}{∥A_{D}^{\prime }r_{\Upsilon^{l}}∥}_{2}$. Using the triangle inequality, (D5) can be rewritten as:

(D6) $$\begin{array}{ccc} ∥A_{D_{K}}^{\prime }r_{\Upsilon_{l}}{∥}_{2} & = & ∥A_{D_{K}}^{\prime }P_{\Upsilon_{l}}^{⊥}{y∥}_{2}\\ & = & ∥A_{D_{K}}^{\prime }P_{\Upsilon_{l}}^{⊥}\left(A_{T}s_{T}+v\right){∥}_{2}\\ & = & ∥A_{D_{K}}^{\prime }P_{\Upsilon_{l}}^{⊥}A_{T\setminus \Upsilon_{l}}s_{T\setminus \Upsilon_{l}}+A_{D_{K}}^{\prime }P_{\Upsilon_{l}}^{⊥}{v∥}_{2}\\ & \leq & ∥A_{D_{K}}^{\prime }P_{\Upsilon_{l}}^{⊥}A_{T\setminus \Upsilon_{l}}s_{T\setminus \Upsilon_{l}}{∥}_{2}+{∥A_{D_{K}}^{\prime }P_{\Upsilon_{l}}^{⊥}v∥}_{2}\\ & = & ∥A_{D_{K}}^{\prime }A_{T\cup \Upsilon_{l}}{\bar{s}}_{T\cup \Upsilon_{l}}{∥}_{2}+{∥A_{D_{K}}^{\prime }P_{\Upsilon_{l}}^{⊥}v∥}_{2}. \end{array}$$

where (D7) is from Definition 2. From (B6) in Appendix B and Lemma 4, (D7) is upper bounded as:

(D7) $$\begin{array}{ccc} ∥A_{D_{K}}^{\prime }r_{\Upsilon_{l}}{∥}_{2} & \leq & ∥A_{D_{K}}^{\prime }A_{T\cup \Upsilon_{l}}{\bar{s}}_{T\cup \Upsilon_{l}}{∥}_{2}+{∥A_{D_{K}}^{\prime }P_{\Upsilon_{l}}^{⊥}v∥}_{2}\\ & \leq & \delta_{2K-i}∥{\bar{s}}_{T\cup \Upsilon_{l}}{∥}_{2}+{∥A_{D_{K}}^{\prime }P_{\Upsilon_{l}}^{⊥}v∥}_{2}\\ & \leq & \delta_{2K-i}∥{\bar{s}}_{T\cup \Upsilon_{l}}{∥}_{2}+∥A_{D_{K}}^{\prime }{∥}_{2}{∥P_{\Upsilon_{l}}^{⊥}v∥}_{2}\\ & \leq & \delta_{2K-i}∥{\bar{s}}_{T\cup \Upsilon_{l}}{∥}_{2}+\sqrt{1+\delta_{|D_{K}|}}{∥P_{\Psi_{l}}^{⊥}v∥}_{2}\\ & \leq & \delta_{2K-i}∥{\bar{s}}_{T\cup \Upsilon_{l}}{∥}_{2}+\sqrt{1+\delta_{\left|D\right|}}{∥v∥}_{2}\\ & \leq & \delta_{2K-i}∥{\bar{s}}_{T\cup \Upsilon_{l}}{∥}_{2}+\sqrt{1+\delta_{K-i}}{∥v∥}_{2}. \end{array}$$

Then by combining (D5) and (D8), we get the desired result as:

(D8) $$\begin{array}{ccc} \alpha^{l} & \leq & \frac{1}{\sqrt{|D_{K}|}}\left[\delta_{2K-i}∥{\bar{s}}_{T\cup \Upsilon_{l}}{∥}_{2}+\sqrt{1+\delta_{K-i}}{∥v∥}_{2}\right]\\ & < & \frac{1}{\sqrt{K-i}}\left[\delta_{2K-i}∥{\bar{s}}_{T\cup \Upsilon_{l}}{∥}_{2}+\sqrt{1+\delta_{K-i}}{∥v∥}_{2}\right] \end{array}$$

Since 1≤i≤K−1, we obtain the desired result.

## Appendix E. Proof of Theorem 6

If the support has the minimum residual in magnitude among all possible candidates with cardinality *K*, then:

$$∥r_{T}{∥}_{2}<{∥r_{\Lambda_{K}}∥}_{2}$$

for any $\Lambda_{K}\neq T$. Note that the above inequality always holds if the upper bound of $∥r_{T}{∥}_{2}$ is smaller than the lower bound of $∥r_{\Lambda_{K}}{∥}_{2}$. In this regard, we first obtain the upper bound of $∥r_{T}{∥}_{2}$ as:

(E1) $$\begin{array}{ccc} ∥r_{T}{∥}_{2} & = & ∥P_{T}^{⊥}{y∥}_{2}={∥P_{T}^{⊥}\left(A_{T}s_{T}+v\right)∥}_{2}\\ & = & ∥P_{T}^{⊥}{v∥}_{2}\leq {∥v∥}_{2} \end{array}$$

where (E1) is because $P_{T}^{⊥}A_{T}x_{T}=0$.

Next, we obtain the lower bound of $∥r_{\Lambda_{K}}{∥}_{2}$ when $\Lambda_{K}\neq T$. For any $\Lambda_{K}\neq T$, we have:

(E2) $$\begin{array}{ccc} ∥r_{\Lambda_{K}}{∥}_{2} & = & ∥P_{\Lambda_{K}}^{⊥}{y∥}_{2}={∥P_{\Lambda_{K}}^{⊥}\left(As+v\right)∥}_{2}\\ & = & ∥P_{\Lambda_{K}}^{⊥}\left(A_{T\setminus \Lambda_{K}}s_{T\setminus \Lambda_{K}}+v\right){∥}_{2}\\ & = & ∥A_{T\cup \Lambda_{K}}{\bar{s}}_{T\cup \Lambda_{K}}{∥}_{2}-{∥P_{\Lambda_{K}}^{⊥}v∥}_{2} \end{array}$$

where (E3) is from the triangle inequality and Definition 2. Using the result from Lemma 7, (E3) is lower bounded as:

$$\begin{array}{ccc} ∥r_{\Lambda_{K}}{∥}_{2} & = & ∥A_{T\cup \Lambda_{K}}{\bar{s}}_{T\cup \Lambda_{K}}{∥}_{2}-{∥P_{\Lambda_{K}}^{⊥}v∥}_{2} \end{array}$$

(E3) $$\begin{array}{ccc} & \geq & \sqrt{1-\delta_{|T\cup \Lambda_{K}|}}∥{\bar{s}}_{T\cup \Lambda_{K}}{∥}_{2}-{∥P_{\Lambda_{K}}^{⊥}v∥}_{2} \end{array}$$

(E4) $$\begin{array}{ccc} & \geq & \sqrt{1-\delta_{|T\cup \Lambda_{K}|}}∥{\bar{s}}_{T\cup \Lambda_{K}}{∥}_{2}-{∥v∥}_{2} \end{array}$$

(E5) $$\begin{array}{ccc} & \geq & \sqrt{1-\delta_{2K}}∥{\bar{s}}_{T\cup \Lambda_{K}}{∥}_{2}-{∥v∥}_{2} \end{array}$$

where (E3) is from Definition 1, (E4) is because $∥P_{\Lambda_{K}}^{⊥}{v∥}_{2}\leq {∥v∥}_{2}$ and (E5) is from Lemma 1. From (E1) and (E5), we have:

(E6) $$\begin{array}{c} ∥{\bar{s}}_{T\cup \Lambda_{K}}{∥}_{2}\geq \frac{{2∥v∥}_{2}}{\sqrt{1-\delta_{2K}}} \end{array}$$

Using the result from Lemma 7, we additionally have:

(E7) $$\begin{array}{c} ∥s_{T\setminus \Lambda_{K}}{∥}_{2}\geq \frac{\sqrt{2}\left(1+\delta_{K}\right){∥v∥}_{2}}{\sqrt{\left(1-\delta_{2K}\right)\left(1+\delta_{K}^{2}\right)}} \end{array}$$

and since $∥s_{T\setminus \Lambda_{K}}{∥}_{2}\geq \min_{j\in T}\left|s_{j}\right|$, we have the desired result.
